# Endoscopic Spacer-Assisted Stereotactic Body Radiation Therapy for Abdominal Oligometastases: A Novel Technique to Reduce Gastrointestinal Toxicity

**DOI:** 10.1016/j.adro.2025.101975

**Published:** 2025-12-06

**Authors:** Sasha Ebrahimi, Jonathan Pham, Albert J. Chang

**Affiliations:** Department of Radiation Oncology, University of California, Los Angeles, California

## Introduction

Spacing agents have emerged as valuable tools in radiation oncology for reducing dose to adjacent critical structures. This is particularly relevant when tumors are close to radiosensitive organs, limiting the feasibility of curative radiation therapy (RT). Initially developed to mitigate rectal toxicity during prostate radiation, commercially available polyethylene-glycol hydrogel (SpaceOAR), hyaluronic acid (Barrigel) and biodegradable balloon spacers (BioProtect), create a temporary physical separation between the prostate and the rectum, thereby reducing radiation dose to the rectal wall.^1^, ^2^, ^3^, ^4^ Spacing agents have also been used in primary radiation treatment of pancreatic,^5^ head and neck,^6^ liver,^7^ and retroperitoneal sarcomas.^8^ Notably, the first-in-human feasibility trial of endoscopic ultrasound guided hydrogel placement in the pancreaticoduodenal groove of 6 patients undergoing stereotactic body radiation therapy (SBRT) demonstrated both procedural safety and stability of the injected material, establishing an important foundation for expanding endoscopic spacer applications to other anatomic regions.^5^ This report describes the novel endoscopic delivery of a hydrogel spacing agent to facilitate safe radiation treatment of a recurrent cervical squamous cell carcinoma (SCC) in the abdomen.

## Clinical Course

A 74-year-old female with recurrent cervical SCC presented with disease progression in the abdomen requiring radiation. Her comorbidities included undifferentiated connective tissue disorder, hypogammaglobulinemia, hypothyroidism, and Parkinson’s disease. The patient also had a history of left breast invasive ductal carcinoma treated approximately 1.5 years prior with breast conserving surgery as well as a supracervical hysterectomy for fibroids approximately 9 years prior to this presentation.

The patient’s initial diagnosis of cervical SCC, staged FIGO IB3, was 4.5 years prior to the recurrence. Magnetic resonance imaging (MRI) of pelvis revealed an enhancing soft tissue mass arising from the cervix measuring up to 3.6 × 4.4 cm with abutment of bladder and sigmoid colon without invasion. Patient was managed with a combination of external beam RT, high-dose rate brachytherapy, and concurrent cisplatin chemotherapy. She received 45 Gy in 25 fractions to the pelvis up to the level of L4 with 5 cycles of concurrent weekly cisplatin (40 mg/m^2^) followed by 25.5 Gy interstitial high-dose rate boost delivered in 3 implants. She subsequently received 3 cycles of adjuvant carboplatin (target area under the curve (AUC) of 5) and gemcitabine (800 mg/m^2^) completed 4 years prior to recurrence. Her follow-up imaging revealed complete resolution of the cervical mass within 9 months of treatment.

**Figure 1:**
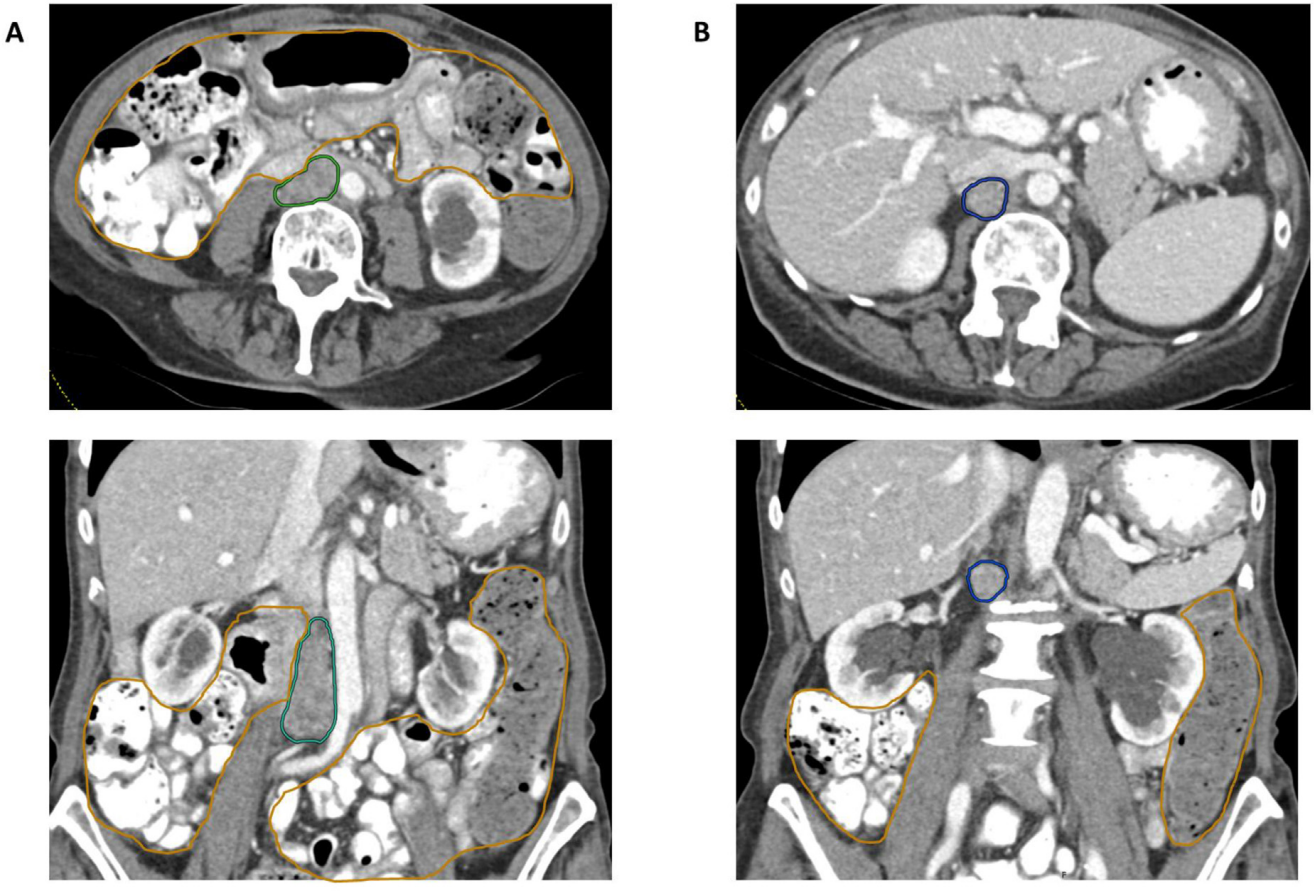
Computed tomography (CT) imaging of 2 pathologically enlarged abdominal lymph nodes concerning for disease progression and recurrence. (A) Axial and coronal intravenous (IV) and oral contrast-enhanced CT images of the abdomen and pelvis show an enlarging aortocaval lymph node (LN1), outlined in green, abutting the duodenum and measuring 18 × 19 × 35 mm, concerning for disease progression. (B) Axial and coronal contrast-enhanced CT images demonstrate an enlarged retroperitoneal lymph node (LN2), outlined in blue and measuring 13 × 15 × 18 mm, confirmed as recurrent cervical squamous cell carcinoma on biopsy. Adjacent bowel loops are outlined in orange.

The patient was diagnosed with screen-detected intraductal carcinoma of the left breast approximately 18 months prior to her recurrence and was treated with lumpectomy and adjuvant letrozole. At the time of presentation, her cervical cancer had progressed, with imaging revealing an enlarging 35-mm aortocaval lymph node (LN1), a right retroperitoneal lymph node (LN2) measuring 12 mm, and a persistent 16-mm right pelvic sidewall mass—all located outside of the prior radiation field (Fig. 1A and B). Biopsy of the LN2 confirmed poorly differentiated SCC, whereas the right pelvic sidewall mass was negative for malignancy. The recurrent cervical SCC exhibited PD-L1 CPS 11, high tumor mutational burden (20), microsatellite stability, an *STK11* deletion, and mutations in *ATR* and *PIK3CA*. Given this pattern of progression, the patient was scheduled to receive radiation to LN1 and LN2, in combination with systemic therapy consisting of carboplatin (AUC 5), paclitaxel (135 mg/m²), pembrolizumab (200 mg), and bevacizumab (15 mg/kg; held during radiation), every 4 weeks.

Patient underwent upper gastrointestinal (GI) endoscopy with endoscopic ultrasound under general anesthesia with an initial plan to place brachytherapy catheters into LN1. Although the paraortic mass was successfully visualized and sampled with fine-needle aspiration, endoscopically guided transduodenal catheter placement was unsuccessful due to inadequate catheter rigidity and a lax bowel wall, which prevented bowel wall penetration. As a result, the treatment strategy was revised to external beam RT with endoscopic placement of a spacing agent via a 19G needle to displace the adjacent bowel from the paraortic lesion.

**Figure 2:**
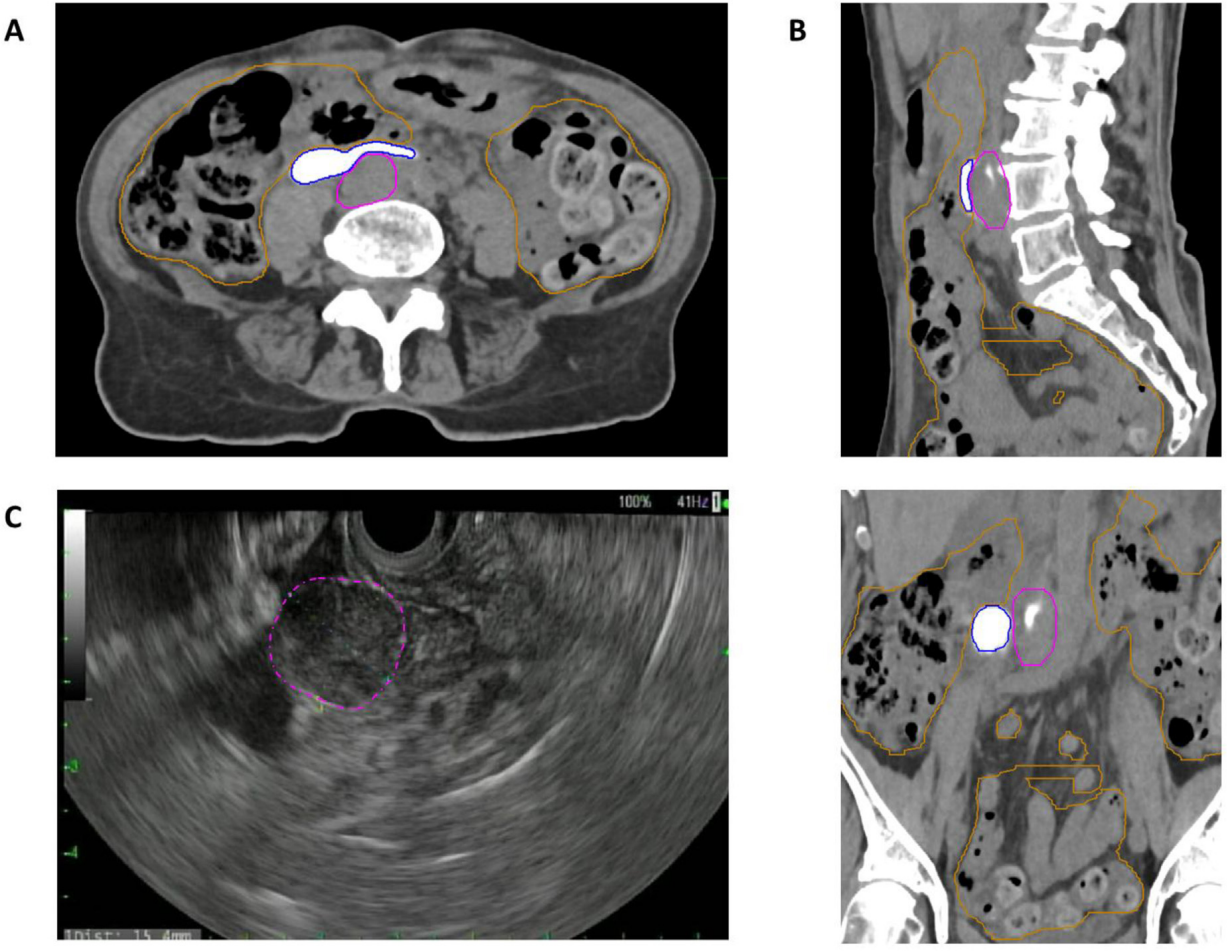
Multimodality imaging demonstrating successful endoscopic placement of a hydrogel spacer (SpaceOAR) anterior to aortocaval lymph node (LN1) to physically displace adjacent bowel structures. (A) Axial, (B) sagittal and coronal noncontrast computed tomography (CT) images confirm the radio-opaque spacer (outlined in blue) positioned between the lymph node (outlined in pink) and adjacent bowel loops (outlined in orange). The hydrogel spacer measured 9.27 cc (dimensions: 5.3 × 3.0 × 1.3 cm). (C) Endoscopic ultrasound (EUS) image obtained during spacer injection shows LN1 (dashed pink contour) prior to hydrogel instillation.

Initial injection of the hyaluronic acid spacer was unsuccessful due to its high viscosity, which prevented delivery through a 19G needle. A radio-opaque polyethylene-glycol-based hydrogel spacer, commercially available as SpaceOAR, was then successfully administered, achieving a separation volume of 9.27 cc (5.3 × 3.0 × 1.3 cm; Fig. 2). The procedure was well tolerated, with no complications or blood loss. The placement of the hydrogel spacer did not compromise target visualization on either MRI or computed tomography (CT). The paraortic lymph node and spacer interface remained clearly delineated on all postplacement imaging, with the spacer appearing as a well-defined, radiopaque structure on CT and hyperintense on T2-weighted magnetic resonance (MR) sequences. During the endoscopic procedure, transient microbubbles were observed on initial injection, but these dissipated within seconds and did not obscure endoscopic ultrasound guidance. No evidence of spacer leakage or unintended hydrogel spread into the retroperitoneal space was observed on procedural or follow-up imaging.

The patient underwent 5-fraction, 36.25-Gy MR guided adaptive SBRT using the ViewRay LINAC system, delivered over 9 days with intrafraction real-time 2-dimensional MR cine tracking and breath-hold respiratory gating. Both LN1 and LN2 were included in the treatment volume. Therapy was administered every other day, following a minimum 3-hour NPO (nil per os) protocol prior to each session. During the treatment course, the patient developed a COVID-19 infection and experienced grade 1 diarrhea and grade 2 fatigue but otherwise tolerated her treatment well. Patient’s COVID-19 infection did not impact her ability to perform breath-hold.

For LN1, a 3-mm margin was added to the gross tumor volume to generate the planning target volume (PTV). Organ-at-risk (OAR) constraints were prioritized over full PTV coverage.^9^^,^^10^ Table 1 summarizes the achieved nominal MR guided plan delivered after endoscopic spacer placement. Only the first fraction required on-table adaptation; all subsequent fractions used this reference plan.

**Table 1:** Plan summary report for MR guided SBRT delivered to LN1

Structure	Rx	Plan
PTV	V95% at 36.25 Gy	92.94%, mean 38.88 Gy, max 42.12 Gy
Duodenum	V33 Gy ≤ 0.5 cc	0.5 cc, mean 12.69 Gy, max 37.29 Gy
Stomach	V33 Gy ≤ 0.5 cc	0 cc, mean 4.41 Gy, max 11.94 Gy
Small bowel	V33 Gy ≤ 0.5 cc	0 cc, mean 5.78 Gy, max 27.01 Gy
Large bowel	V33 Gy ≤ 0.5 cc	0 cc, mean 1.56 Gy, max 8.66 Gy
Liver	V15 Gy ≤ 700 cc, mean ≤ 20 Gy	31.88 cc, mean 5.17 Gy, max 38.63 Gy
Cord	V25 Gy ≤ 0.5 cc	0 cc, mean 4.99 Gy, max 9.57 Gy
Right kidney	V14 Gy ≤ 33%, mean ≤ 12 Gy	0%, mean 3.85 Gy, max 10.44 Gy
Left kidney	V14 Gy ≤ 33%, mean ≤ 12 Gy	0%, mean 2.87 Gy, max 6.99 Gy

*Abbreviations:* max = maximum; LN1 = aortocaval lymph node; MR = magnetic resonance; Rx = prescribed dose constraints; SBRT = stereotactic body radiation therapy.

The table compares prescribed dose constraints to the achieved plan dosimetry.

**Figure 3:**
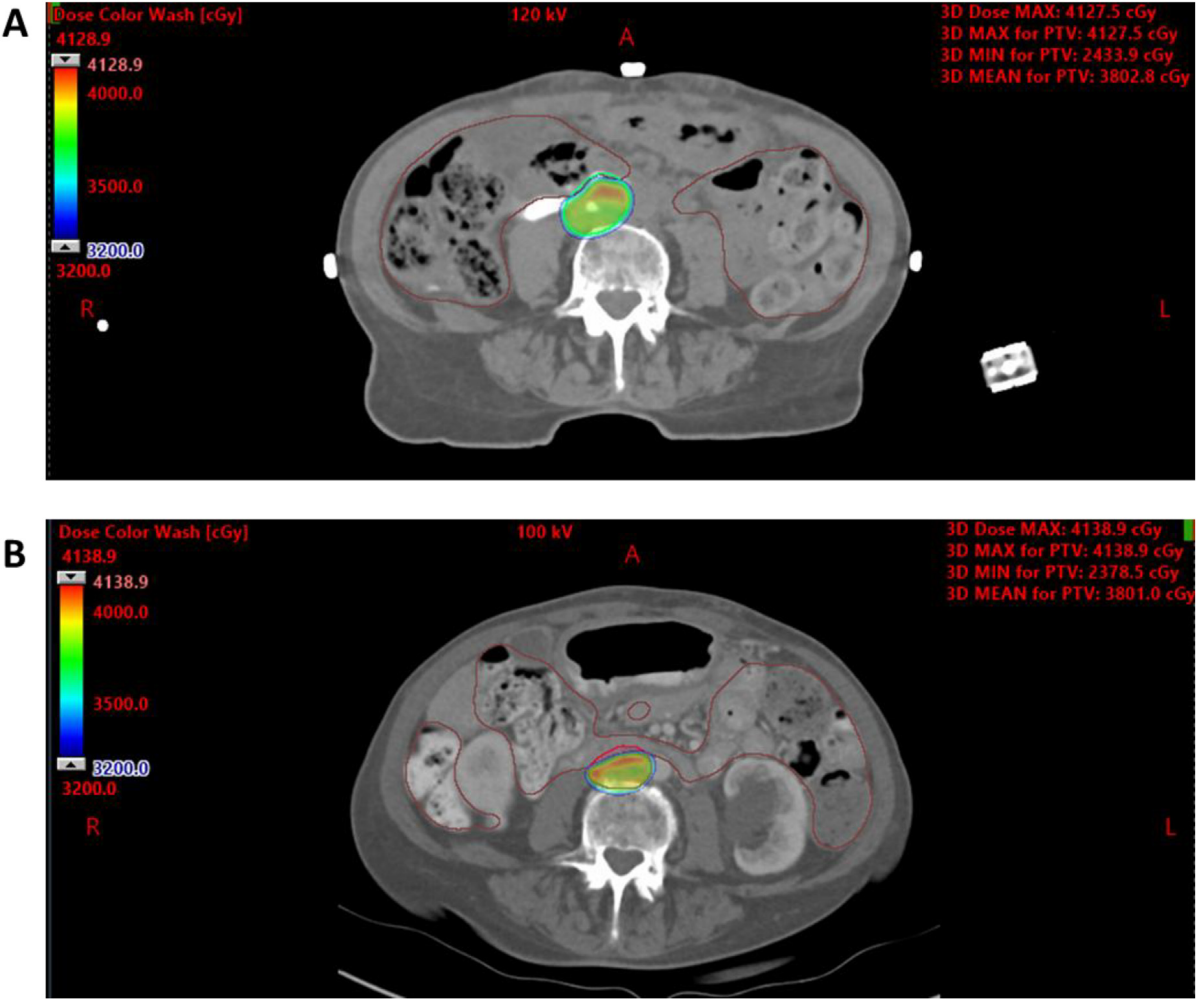
Stereotactic body radiation therapy (SBRT) planning comparison highlighting compromised planning target volume (PTV) coverage when bowel constraints are prioritized in the absence of a hydrogel spacer. Axial contrast-enhanced computed tomography (CT) images with overlaid SBRT dose color wash (range, 3200-4138.9 cGy) illustrate treatment planning for aortocaval lymph node (LN1) using the same target contours, comparing scenarios with and without a hydrogel spacer. (A) and (B) show axial slices from plans generated using a Novalis Tx LINAC with HD120 MLC. The high-dose region (green to red) reflects the prescription dose distribution relative to the PTV, outlined in red. In the absence of the spacer and with bowel organ-at-risk (OAR) constraints prioritized, there is visible undercoverage of the PTV due to dose limitation near adjacent bowel loops.

To isolate the dosimetric effect of spacer placement alone, we created in-silico comparative SBRT plans using pre- and postspacer CT scans, using the Novalis Tx LINAC with HD120 MLC, which is the standard platform used for all CT-based SBRT planning at our institution. When prioritizing the AAPM TG-101 D0.035cc constraint of ≤32 Gy to the duodenum (achieving 31.85 Gy with SpaceOAR vs 31.97 Gy without), we attained 90% PTV coverage with the spacer compared to only 84% without it^11^ (Table 2). This trade-off is illustrated in Fig. 3, where the SBRT plan without the spacer shows undercoverage of the PTV, with portions of the target volume extending beyond the high-dose color wash. Conversely, when prioritizing 90% PTV coverage without the spacer, the plan violated duodenal constraints, with the D0.035cc reaching 35.23 Gy. Different OAR constraints were applied between the MR guided delivered plan and the CT-based in-silico plans because SMART trial limits (D0.5cc < 33 Gy)^10^ are clinically accepted for MRI-adaptive SBRT with daily online replanning, whereas TG-101 is intentionally more conservative for nonadaptive, CT-based SBRT planning.

**Table 2:** Dosimetric comparison of SBRT treatment plans with and without SpaceOAR spacer placement, evaluating target coverage and OAR sparing

Structure	Rx	With SpaceOAR	No SpaceOAR PTV prioritized	No SpaceOAR OARs prioritized
PTV	V95% at 36.25 Gy	90%	90%	84%
Stomach	D0.035 cc ≤ 32 Gy	14.12 Gy	11.65 Gy	11.64 Gy
Duodenum/jejunum	D0.035 cc ≤ 32 Gy	31.85 Gy	35.23 Gy	31.97 Gy
Liver	MVS21 Gy ≥ 700 cc	1262 cc	1085 cc	1085 cc
Renal cortex	MVS17.5 Gy ≥ 200 cc	254 cc	221 cc	221 cc
Bladder wall	D0.035 cc ≤ 38 Gy	0.04 Gy	0.06 Gy	0.06 Gy
Cauda equina	D0.035 cc ≤ 32 Gy	12.28 Gy	10.67 Gy	10.92 Gy

Three planning scenarios are shown: with SpaceOAR, without SpaceOAR prioritizing PTV coverage, and without SpaceOAR prioritizing OAR constraints. Prescription goals (Rx) are listed for each structure.

*Abbreviations:* MVS = minimum volume spared; OAR = organ-at-risk; PTV = planning target volume; Rx = prescribed dose constraints; SBRT = stereotactic body radiation therapy.

**Figure 4:**
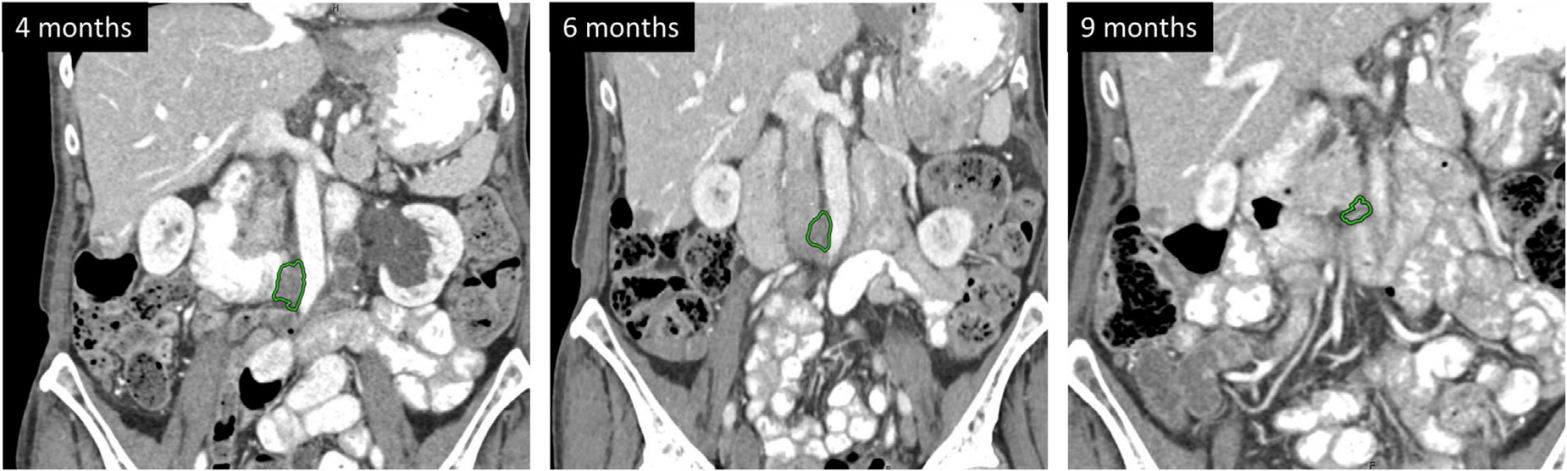
Serial imaging demonstrating treatment response in aortocaval lymph node (LN1) and complete resorption of the hydrogel spacer following stereotactic body radiation therapy (SBRT). Coronal intravenous (IV) and oral contrast-enhanced abdominal computed tomography (CT) images demonstrate a progressive decrease in the size of the radiated aortocaval lymph node, consistent with treatment response. The lymph node measured 18 × 19 × 35 mm pretreatment (Fig. 1A), with subsequent reductions to 9 × 12 × 19 mm at 4 months, 8 × 12 × 11 mm at 6 months, and 7 × 10 × 8 mm at 9 months posttreatment. Additionally, the previously placed SpaceOAR is no longer detectable on posttreatment scans after 4 months, indicating complete resorption.

The patient subsequently completed 6 cycles of carboplatin, paclitaxel, pembrolizumab, and bevacizumab by 6 months postrecurrence. Systemic therapy was intermittently delayed due to immune thrombocytopenic purpura-related thrombocytopenia. She continues on maintenance pembrolizumab and bevacizumab every 3 weeks. Follow-up surveillance imaging demonstrated a favorable treatment response, with a reduction in the size of LN1 from 18 × 19 × 35 mm pretreatment to 7 × 10 × 8 mm at 9 months post-SBRT (Fig. 4). A positron emission tomography/CT scan performed 1 year after treatment showed no evidence of local or distant disease recurrence. There was no reported late radiation-related toxicity after 1 year posttreatment.

## Discussion

This case highlights the expanding role of spacing agents beyond their established use in prostate cancer RT. Although they are well-recognized for mitigating rectal toxicity during prostate RT, their application in abdominal and retroperitoneal tumors remains less documented but equally important. Several early-stage studies have demonstrated the utility of spacers in these regions. In locally advanced pancreatic cancer, surgical spacer placement has been shown to improve target coverage and spare adjacent organs by creating meaningful separation, with favorable early outcomes.^12^^,^^13^ Similarly, laparoscopic spacer placement has enabled safe radiation delivery to bulky aortocaval nodal recurrences of cervical cancer by displacing the duodenum and facilitating dose escalation.^14^ Although endoscopic ultrasound guided hydrogel injection in the pancreaticoduodenal groove has been previously demonstrated in a prospective pilot study for locally advanced pancreatic cancer,^5^ this case represents the first clinical application of endoscopic spacer placement for a nonpancreatic abdominal oligometastasis treated with MR guided SBRT. The distinction underscores the feasibility of translating this technique beyond pancreatic applications. At last follow-up, the patient had stable disease with no late GI toxicity.

In this patient, the aortocaval lymph node’s proximity to bowel posed a significant challenge for delivering safe and effective radiation. Meeting OAR constraints is essential in abdominal RT due to the frequent adjacency of tumors to sensitive structures such as the bowel, stomach, and liver. Spacing agents offer a practical solution by physically displacing these organs, thereby reducing the risk of severe toxicity—including bowel perforation, gastrointestinal bleeding, strictures, and chronic pain. These considerations are increasingly relevant as the field shifts toward higher biologically effective dose (BED), hypofractionated SBRT regimens. For instance, late grade ≥ 3 GI toxicity associated with pancreatic SBRT is estimated to be between 7% and 13% with GI ulceration as the predominant serious toxicity.^15^^,^^16^ While advanced technologies like MRI guidance, intrafraction monitoring, and respiratory gating can improve OAR sparing, they are costly and not universally available.

This case contributes to the growing body of evidence supporting the versatility of spacing agents in improving the therapeutic ratio for anatomically complex targets. Future prospective studies should evaluate spacer placement techniques (endoscopic vs laparoscopic), timing, and long-term clinical impact.

## Disclosures

Albert J. Chang reports a relationship with Elekta, Boston Scientific, and BioProtect Ltd that includes consulting or advisory and speaking and lecture fees. The other authors declare that they have no known competing financial interests or personal relationships that could have appeared to influence the work reported in this paper.
